# Supplementary material for: Correlative Fluorescence- and Electron Microscopy of Whole Breast Cancer Cells Reveals Different Distribution of ErbB2 Dependent on Underlying Actin
Source: Front Cell Dev Biol. 2020 Jun 30;8:521. doi: 10.3389/fcell.2020.00521 (PMC7344305; doi:10.3389/fcell.2020.00521)
Supplement: FIGURE S1 — Light microscopy of Actin- and ErbB2 stained SKBR3 cell displayed in Figure 2 of the main text to indicate membrane ruffles. (A) Peripheral ruffles appear as darker gray structures at the cell edge in the direct interference contrast (DIC) image. (B) Actin-green fluorescent protein (GFP) gives a strong signal at the same positions. (C) Quantum dot (QD)-stained ErbB2 molecules overlay with actin-containing peripheral ruffles. (D) Overlay of the DIC, GFP and QD-channel as displayed in (A–C). [file Data_Sheet_1.PDF]

Supplementary Information

Correlative Fluorescence and Electron Microscopy of Whole Breast Cancer Cells Reveals Different Distribution of ErbB2 Dependent on Underlying Actin

Indra Navina Dahmke, Patrick Trampert, Florian Weinberg, Zahra Mostajeran, Franziska Lautenschläger and Niels de Jonge

Supplementary Figures

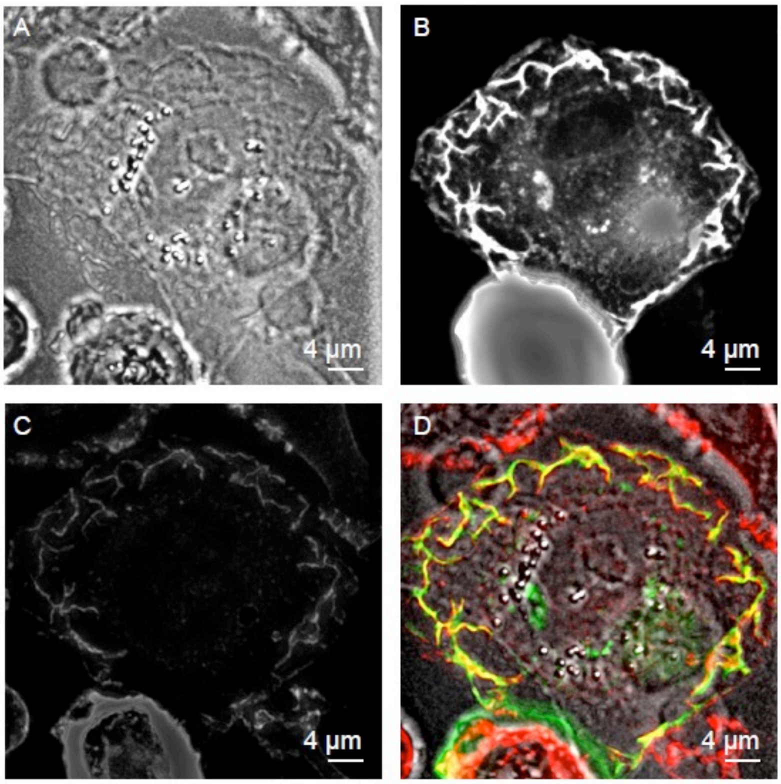

Figure S1. Light microscopy of Actin- and ErbB2 stained SKBR3 cell displayed in Figure 2 of the main text to indicate membrane ruffles. **(A)** Peripheral ruffles appear as darker grey structures at the cell edge in the direct interference contrast (DIC) image. **(B)** Actin-green fluorescent protein (GFP) gives a strong signal at the same positions. **(C)** Quantum dot (QD)-stained ErbB2 molecules overlay with actin-containing peripheral ruffles. **(D)** Overlay of the DIC, GFP and QD-chanel as displayed in A-C.

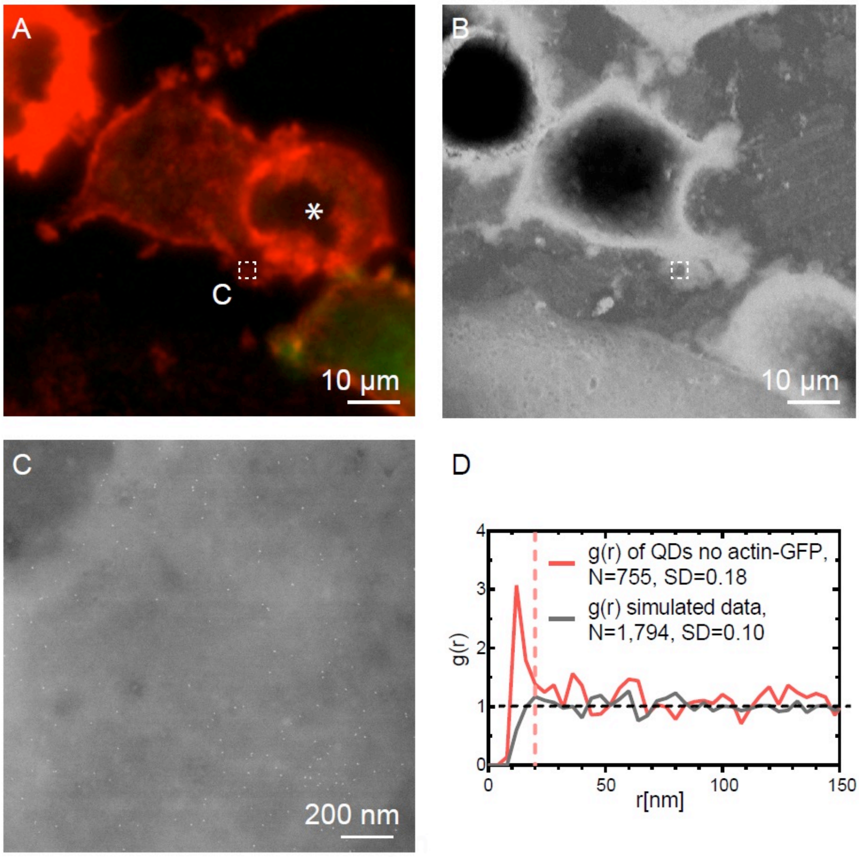

Figure S2. ErbB2 stained SKBR3 cell without actin-GFP. **(A)** Cropped fluorescence micrograph of SKBR3 breast cancer cells showing QD-labeled membrane ErbB2 in red. Cell marked with * was lost during EM preparation process. **(B)** Corresponding low magnification scanning transmission electron micrograph of graphene covered breast cancer cells taken at the same area, Magnification M = 1000×. **(C)** High resolution scanning transmission electron (STEM) micrographs of region marked in a-b. QD-labels appear as white dots, M = 100,000×. **(D)** Pair correlation function $g(r)$ as function of pair distance r of QD-labeled ErbB2 in flat regions without actin-GFP.

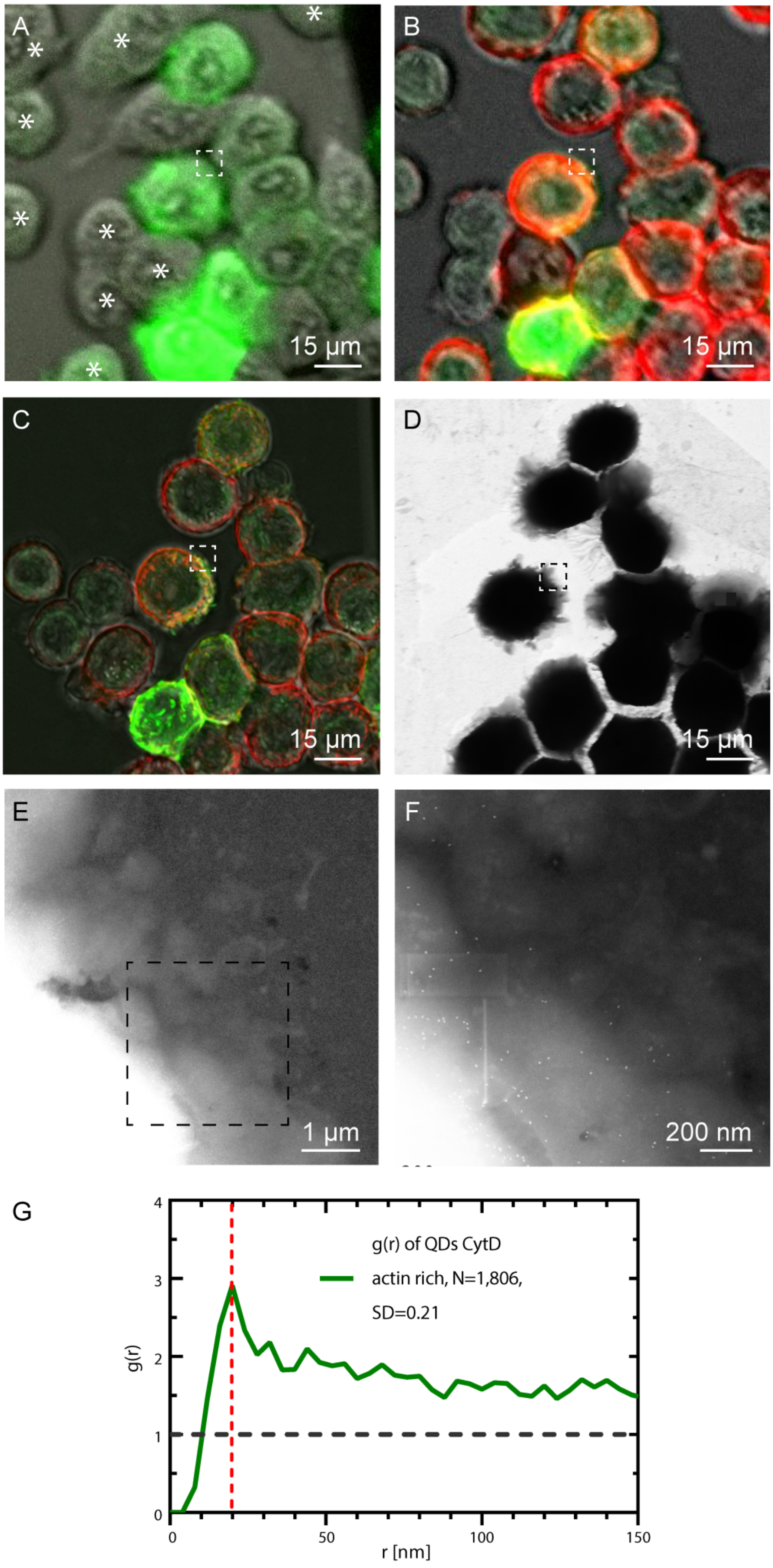

A
15 µm
B
15 µm
C
15 µm
D
15 µm
E
1 µm
F
200 nm
G
g(r) of QDs CytD
actin rich, N=1,806,
SD=0.21
g(r)
r [nm]
0
1
2
3
4
0
50
100
150

Figure S3. Correlative fluorescence microscopy and STEM of whole breast cancer cells after treatment with Cytochalasin D (Cyt D). **(A-C)** Cropped fluorescence micrographs of SKBR3 breast cancer cells showing cellular actin-GFP in green, and QD-labeled membrane ErbB2 in red. Areas were both signals overlay appears yellow. Image A was taken at the baseline before treatment with Cytochalasin D (1h, 2μM) and staining of QDs. Image B was acquired immediately after Cytochalasin D treatment and labeling of ErbB2 with QDs. Image C was generated of maximum intensity overlays of deconvoluted z-stacks from the same spot as in A and B. Cells that disappeared during Cytochalasin D treatment and EM preparation process are marked with white asterisks in image A. **(D)** Corresponding low magnification scanning transmission electron micrograph of graphene covered breast cancer cells taken at the same area, $M = 1000\times$. **(E, F)** High resolution scanning transmission electron micrographs of region marked in a-d. QD-labels appear as white dots in F. E: $M = 30{,}000\times$, F: $M = 100{,}000\times$. (G) Graph of pair correlation function $g(r)$ as function of pair distance r of QD-labeled ErbB2 in peripheral cellular regions containing actin-GFP as displayed in (A-F). Note the peak at 20 nm which is similar to the one of actin-rich areas in untreated cells.

**Experimental group**	**No. of experiments**	**No. of cells**	**No. of images**	**No. of all particles**	**Total area (μm^2)**	**Average particle density per image (particles/μm^2)**
**Cyt D treated cells, actin rich areas**	3	8	12	1,806	46	39

Supplement Table 1. Summary of scanning transmission electron microscopy (STEM) data used for label distribution analysis of STEM images acquired at actin-rich regions of Cytochalasin D (Cyt D) treated SKBR3 breast cancer cells that were transduced with actin-green fluorescent protein (GFP).
